# F-18 Fluciclovine PET-CT Findings and Pseudoprogression on Immunotherapy

**DOI:** 10.7759/cureus.30380

**Published:** 2022-10-17

**Authors:** Bradley C Poindexter, Nandita M Kasireddy, Olga P Molchanova-Cook

**Affiliations:** 1 Radiology, Burrell College of Osteopathic Medicine, Las Cruces, USA; 2 Radiology, Dartmouth College, Hanover, USA; 3 Radiology, Memorial Medical Center, Las Cruces, USA

**Keywords:** immunotherapy radiological findings, cancer immunotherapy, castration-resistant metastatic prostate cancer, pet-ct fluciclovine, pseudo-progression

## Abstract

Immunotherapy plays a vital role in the treatment of several types of malignancies. While the molecular targets of immunotherapy differ, the desired goal is to increase the host immune response against neoplastic tissue. This upregulated immune state results in infiltration of the tumor with activated immune cells and may be misinterpreted as disease progression in anatomical and metabolic imaging studies, known as pseudoprogression. We present a case of pseudoprogression demonstrated on a fluoride-18 (F-18) fluciclovine positron emission tomography-computed tomography (PET-CT) scan of an 85-year-old male with metastatic castrate-resistant prostate cancer who underwent treatment with sipuleucel-T. An understanding of the pseudoprogression phenomenon and its manifestations is critical for both treating physicians and imaging specialists to facilitate decision-making regarding treatment.

## Introduction

In the treatment of castrate-resistant prostate cancer, chemotherapy remained the most commonly utilized therapy until recently. Given the significant, associated side effects, other therapeutic options are being developed, including immunotherapy and bone-targeting treatments. The effects of immunotherapy take time to develop, making it difficult for a physician to know if the patient is benefitting from the treatment. In the evaluation of treatment response, various imaging modalities are used. Metabolic imaging utilizing positron emission tomography-computed tomography (PET-CT) is one of the most prolific tools used across all types of cancer. Imaging findings remain one of the criteria used to assess treatment response. Therefore, an understanding of how to differentiate between pseudoprogression, caused by the increased immune response induced by immunotherapy, and true progression is important for imaging specialists and oncologists.

## Case presentation

**Figure 1:**
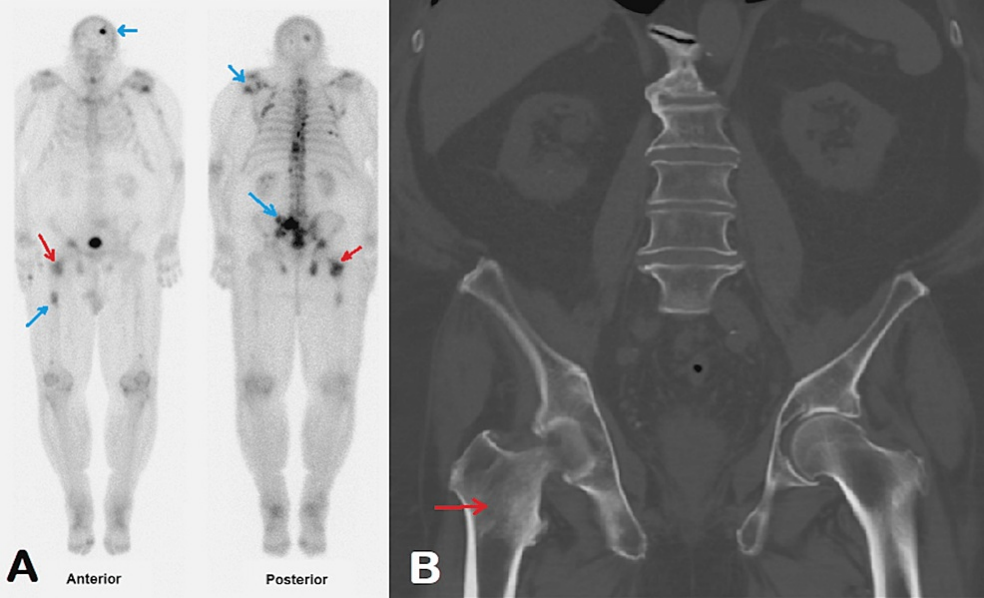
Findings on baseline imaging in October 2020 (A) Technetium-99m MDP whole-body bone scan shows multifocal abnormal radiotracer uptake in the skeleton (blue arrows), compatible with diffuse osseous metastatic disease; (B) CT without intravenous contrast shows multiple sclerotic lesions in the skeleton, compatible with bone metastases. The sclerotic lesion in the right intertrochanteric region on CT shows corresponding bone scan uptake (red arrows). MDP - methylene diphosphonate

We are presenting a case of an 85-year-old male with metastatic Stage IV castrate-resistant prostatic adenocarcinoma Gleason score of 9 (grade 4+5), initially diagnosed in September 2020. Prostate-specific antigen (PSA) at the time of diagnosis was 13. The patient had an additional past medical history of chronic kidney disease, diabetes mellitus, and hypertension. A whole-body bone scan in October 2020 showed multifocal osseous metastatic disease corresponding to sclerotic lesions on concurrent CT (Figure 1). CT in October 2020 also showed enlarged prostate and enlarged pelvic lymph nodes, likely metastatic. The patient was started on enzalutamide 160 mg PO daily (nonsteroidal antiandrogen medication) in December 2020 and continues currently, and denosumab 120 mg subcutaneously every four weeks (bone-modifying agent). The patient is also on continued androgen suppression therapy with leuprolide acetate injections once a month since the initial diagnosis.

In October 2021, the patient reported worsening nausea and an 11-pound weight loss. PSA levels also started to rise; PSA in October 2021 was 0.82, which increased from 0.35 in September 2021. To better evaluate the extent of the disease, a fluoride-18 (F-18) ﬂuciclovine PET-CT scan was performed. F-18 fluciclovine is a synthetic, non-metabolized amino acid analog PET radiotracer that was approved by the Food and Drug Administration (FDA) for the evaluation of patients with suspected prostate cancer recurrence based on elevated PSA levels following prior treatment. The standard departmental F-18 fluciclovine imaging protocol was followed for imaging[1]. The patient was placed in the PET-CT scanner and injected with 10.0 mCi of F-18 fluciclovine intravenously. The acquisitions were obtained approximately four minutes after radiotracer administration from the top of the upper thighs to the mid skull. CT images were used primarily for attenuation correction and localization. The exam was performed according to our departmental dose-optimization program, which includes automated exposure control and adjustment of the mA and/or kV according to patient size. Standardized uptake values (SUVs) were reported as SUVmax (maximum value in the region of interest). Measured SUVs will vary with time between radiotracer injection and imaging, acquisitions/processing parameters, and patient weight. For these reasons, special effort is usually made to maintain imaging parameters constant between different studies to allow the use of changes in SUV to evaluate metabolic activity in the tumor.

**Figure 2:**
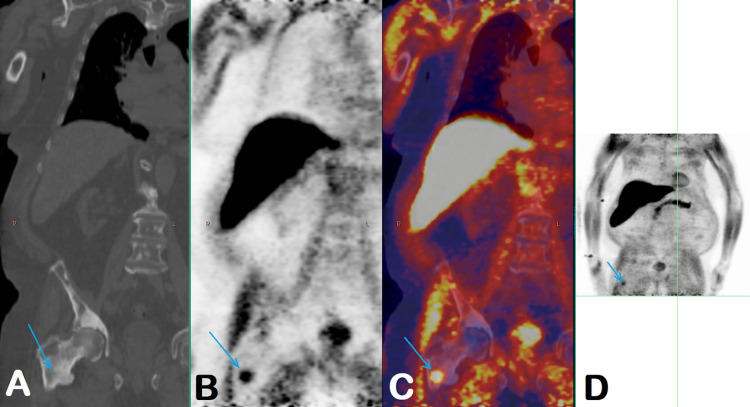
Findings on the fluoride-18 fluciclovine PET-CT scan in October 2021 CT (A), PET (B), fused PET-CT (C), and MIP (D) images show intense focal radiotracer activity in the intertrochanteric region of the right femur (blue arrows), not in the prior sclerotic lesion, but in the adjacent, less dense sclerotic area. It shows SUVmax of 4.1 (for reference, background physiologic bone marrow activity SUVmax of 2.5). PET – positron emission tomography, MIP – maximum intensity projection, SUVmax – maximum standardized uptake value

A fluoride-18 fluciclovine PET-CT scan in October 2021 showed heterogeneous, abnormally increased activity in the prostate, highly concerning for viable neoplasm. Several foci of abnormal radiotracer activity were seen in the skeleton with corresponding sclerotic lesions, likely representing active osseous metastases (Figure 2). But not all sclerotic lesions showed abnormal radiotracer uptake, which could represent treated non-viable lesions [2]. The overall number and extent of sclerotic lesions increased compared to the prior CT and bone scan in October 2020. Enlarged pelvic lymph nodes seen on the prior CT from October 2020 resolved in the interim. There was no other imaging performed between October 2020 (initial diagnosis) and October 2021 (recurrence).

The patient was referred to a subspecialty urological oncology center for evaluation of eligibility for sipuleucel-T therapy. An alternative option was starting chemotherapy. The patient was found to be eligible for sipuleucel-T therapy and continued treatment with enzalutamide and leuprolide acetate.

Labs in January 2022 prior to treatment with sipuleucel-T showed a PSA of 2.96, white blood count (WBC) of 3,200, absolute neutrophil count (ANC) of 1,630, lymphocyte count of 1,000, and alkaline phosphatase of 91. The patient received sipuleucel-T therapy in February 2022. He also continued therapy with enzalutamide and leuprolide acetate.

At clinical follow-ups in April and May 2022 after treatment with sipuleucel-T, the patient reported having a good appetite and feeling well. He rated both his fatigue and pain levels at 0 out of 10. The Eastern Cooperative Oncology Group (ECOG) performance status was 0. In April 2022, PSA was 6.17. Labs in May 2022 showed a PSA of 10.01, WBC count of 4,400, ANC of 2,470, lymphocyte count of 1,260, and alkaline phosphatase of 155.

**Figure 3:**
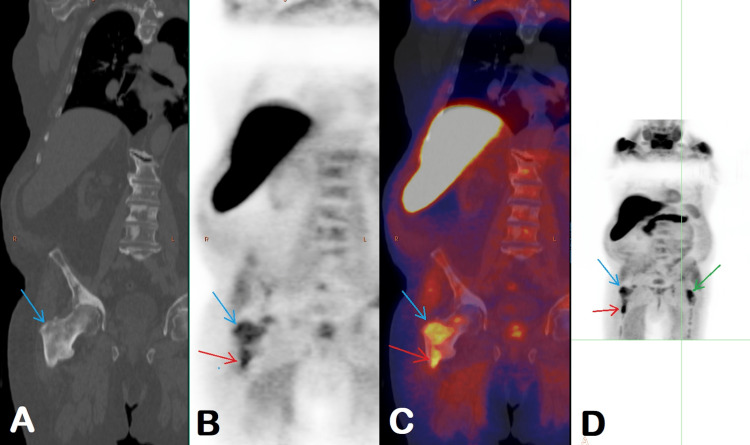
Findings on a fluoride-18 fluciclovine PET-CT scan in April 2022 CT (A), PET (B), fused PET/CT (C), and MIP (D) images show an increased number of foci of abnormally increased radiotracer activity in the skeleton (red arrows). There is an increased extent of sclerosis and associated abnormal radiotracer activity in the intertrochanteric region of the right femur (blue arrows). Activity in the previously seen lesion decreased to a SUVmax of 3.5 from a previous SUVmax of 4.1 (for reference, background bone marrow activity SUVmax of 2.8). Activity in the new area of sclerosis is SUVmax of 5.5. The new focus of abnormal radiotracer uptake was seen corresponding to the faint sclerotic lesion in the left femoral neck (green arrow) with a SUVmax of 5.5. PET – positron emission tomography, MIP – maximum intensity projection, SUVmax – maximum standardized uptake value

A fluoride-18 fluciclovine PET-CT scan in April 2022, two months after sipuleucel-T therapy, showed persistent abnormal activity in the prostate. There were more foci of abnormally increased radiotracer activity in the skeleton than in the previous PET-CT (Figure 3).

In the evaluation of treatment response to immunotherapy, the appearance of new lesions is treated as "unconfirmed progression" and requires imaging follow-up in six to eight weeks. "Confirmed progression" is diagnosed on the follow-up scan if the lesion progresses further. Given the patient’s excellent performance status, findings of increased areas of abnormal radiotracer activity in the skeleton were suspected to represent immunotherapy-related pseudoprogression. A decision was made to continue surveillance and repeat imaging in six to eight weeks.

**Figure 4:**
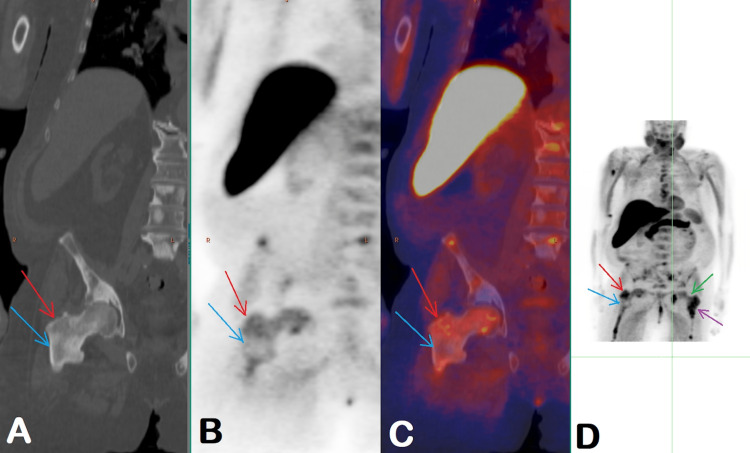
Findings on a fluoride-18 fluciclovine PET-CT scan in June 2022 CT (A), PET (B), fused PET/CT (C), and MIP (D) images show the complete resolution of previously seen abnormal activity in the oldest sclerotic lesion in the intertrochanteric region of the right femur (blue arrows) and decreased intensity of activity in the adjacent sclerotic area (red arrows) with a SUVmax of 4.4 from a previous SUVmax of 5.5 (for reference, background bone marrow activity SUVmax of 2.8). Activity in the previously “hot” lesion in the left hip also decreased (green arrow) with SUVmax of 3.0 from the previous SUVmax of 5.5, but there is new adjacent sclerosis with intense radiotracer activity (purple arrow) with SUVmax 5.5. PET – positron emission tomography, MIP – maximum intensity projection, SUVmax – maximum standardized uptake value

A fluoride-18 fluciclovine PET-CT scan in June 2022, eight weeks after the prior scan and four months after sipuleucel-T therapy showed the development of new foci of abnormally increased radiotracer activity with corresponding new, faintly sclerotic lesions. Several previously present lesions showed decreased associated radiotracer activity (Figure 4). Decreased activity in previously seen lesions, classified as "unconfirmed progression," is suggestive of the phenomenon of pseudoprogression demonstrated in the study of April 2022. However, immunotherapy-related progression was previously described on metabolic imaging with F-18 fluorodeoxyglucose and in solid tumors, not in prostate cancer [3].

On a follow-up visit in early July 2022, the patient’s serum PSA level continued to rise to 29.8 (from 10.01 in May 2022). The patient now complained of new pain in the left lower extremity, had a new weight loss of 5-6 pounds, and decreased appetite. The overall assessment was consistent with progressive disease. The patient was started on chemotherapy with docetaxel.

## Discussion

Immunotherapy has recently proven to be a promising alternative to chemotherapy in the treatment of advanced cancers. However, immunotherapy utilizes multiple, novel pharmaceuticals with diverse mechanisms of action, causing tumors to respond differently than they do to conventional, systemic chemotherapy. One of these differences is the delayed effect that several immunotherapeutic drugs have shown. For example, sipuleucel-T was approved by the FDA after showing improvement in the overall survival of patients with castrate-resistant prostate cancer when compared with a placebo, despite not improving time to disease progression or affecting PSA levels [4]. With its minimal adverse side effects, such as fever and headache, and evidence that it clearly induces a systemic T-cell response within the tumor and in the patient, it still proves a promising treatment, adjunct, and alternative to conventional chemotherapy.

A phase III IMPACT study using sipuleucel-T as a treatment in men with metastatic castration-resistant prostate cancer found additional evidence of the delayed treatment effect on proximal clinical outcomes, not impacting early disease progression and onset of disease-related pain, but improving later outcomes such as overall survival [5]. Interestingly, it also seemed to be more beneficial when given in the early stages of cancer, as well as in African American men versus white men [5]. Given the relatively long natural history of prostate cancer and rising PSA as the initial manifestation of disease progression and clinical worsening, immunotherapy use with its delayed benefits seems to be well suited for the treatment of such cancer. Clearly, the mechanism of sipuleucel-T warrants further investigation given its unique treatment outcomes.

**Figure 5:**
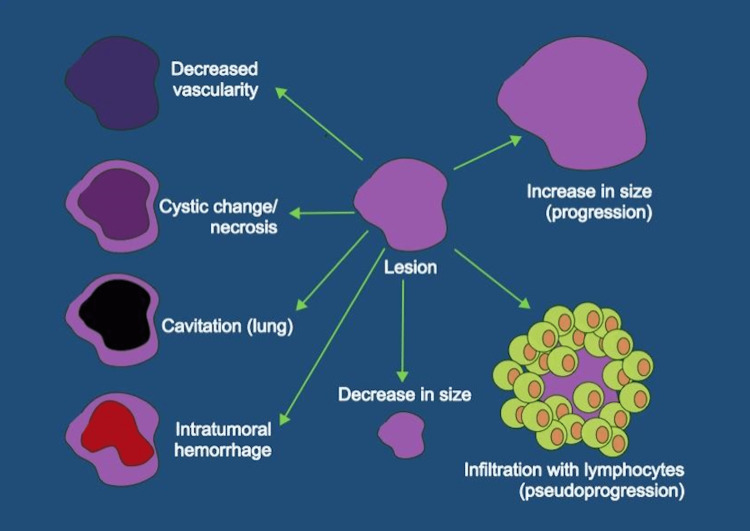
Morphological responses in tumors to different types of cancer treatments Response to chemotherapy is usually evaluated by size change: a decrease in size implies a favorable response, increase in size is progression. Some other changes in the morphological appearance of the tumor could be suggestive of treatment response, including the development of necrosis, cavitation, decreased vascularity/enhancement, or intratumoral hemorrhage. However, with the use of immunotherapy infiltration with immune cells may result in an apparent increase in the size of the lesion and increased activity on metabolic imaging due to the presence of highly active immune cells rather than due to the growth of the tumor, which constitutes pseudoprogression.

Using the disease markers and imaging response assessment criteria that were developed for conventional chemotherapy may often prevent physicians from accurately interpreting responses to treatment when immunotherapy is used (Figure 5) [6]. One example of this is the lack of effect on PSA levels from treatment with sipuleucel-T. Additionally, the increased host immune response triggered by immunotherapy results in the infiltration of the tumor with immune cells resulting in the initial appearance of increased tumor burden, causing pseudoprogression, which is usually followed by a prolonged favorable response. In the 2015 St. Gallen Advanced Prostate Cancer Consensus Conference (APCCC), 82% of the panel stated that if two of three criteria (increasing PSA, worsening clinical status, and evidence of disease progression on imaging), treatment should be stopped or changed [7]. According to the APCCC guidelines, this patient would have met two of the three criteria to stop or change treatment despite PSA levels not being affected by sipuleucel-T and the possibility of pseudoprogression on imaging. Clinicians unfamiliar with the concept of pseudoprogression may not be able to distinguish pseudoprogression from the true progression of the disease, possibly resulting in the cessation of life-saving treatment.

**Figure 6:**
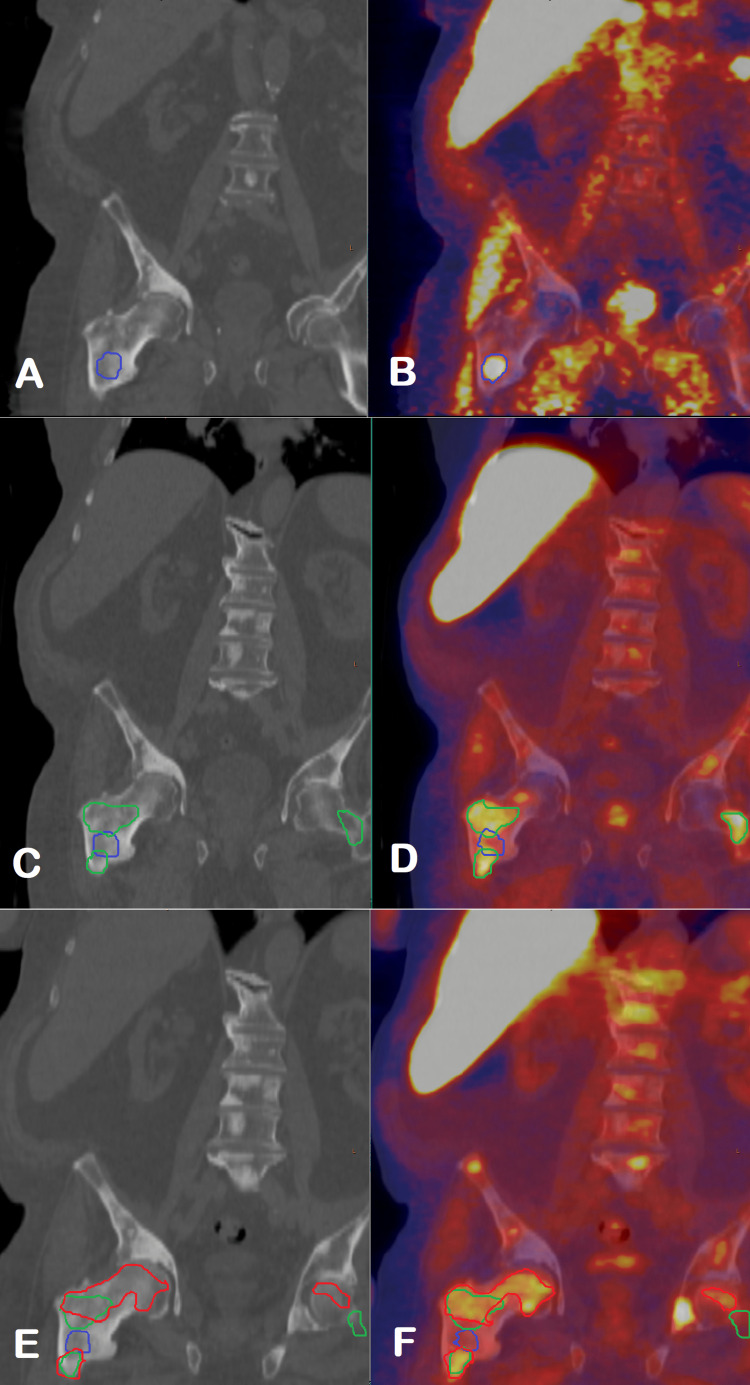
Changes in osseous lesions on Fluoride-18 fluciclovine PET-CT scan after treatment with sipuleucel-T CT (A) and fused PET-CT (B) images from October 2021 prior to sipuleucel-T treatment show an intense focal radiotracer activity in the intertrochanteric region of the right femur, corresponding to a new faintly dense lesion (blue outline), adjacent to a more densely sclerotic non-active lesion, which was present on imaging from October 2020. In the first follow-up study after sipuleucel-T treatment in April 2021, CT (C) and fused PET-CT (D) images show increased extent of sclerosis and associated abnormal radiotracer activity in the intertrochanteric region of the right femur (green outline), although activity in the previously active lesion decreased (blue outline). There is also a new area of faint sclerosis in the left femoral neck with intense radiotracer activity. On the CT (E) and fused PET-CT (F) images from June 2022, there is complete resolution of previously seen abnormal activity in the initially active lesion in the right hip (blue outline), decreased intensity of activity in the adjacent sclerotic area, and in the left femoral neck region (green outline). However, new areas of sclerosis and metabolic activity are seen (red outline).

The patient described here has shown an atypical pattern of response to systemic immunotherapy for the treatment of prostate cancer (Figure 6). After starting immunotherapy, the first follow-up fluoride-18 fluciclovine PET-CT scan in April 2022 showed a decreased intensity of radiotracer activity in the pre-existing osseous lesions, although new metabolically active lesions were seen, mainly closely adjacent to previously detected lesions. In the evaluation of treatment response to immunotherapy in solid tumors, it was found that the appearance of new lesions does not necessarily mean progression, but rather classified as “unconfirmed progression,” which requires imaging follow-up in six to eight weeks. If lesions show continued progression on a follow-up scan, it is classified as “true progression.” If lesions improve, it is classified as evidence of pseudoprogression. Given that the patient was clinically doing very well, this was interpreted as possible pseudoprogression rather than progression. New foci of activity in skeletal lesions were felt to be most likely reflective of uptake in the activated immune cells rather than growing active neoplastic lesions. However, the assumption of pseudoprogression was made based on the previously described findings of increased F-18 fluciclovine uptake in inflammatory and infectious processes similar to those seen on F-18 FDG scans [8]. There is only anecdotal evidence of increased uptake of F-18 fluciclovine in inflammatory lesions, but it warrants further research.

The decision was made to continue surveillance and follow up with imaging in eight weeks. The second follow-up scan in June 2022 showed complete resolution of activity in the initially “hot” right hip lesion and decreased activity in the lesions, which were “new” on the prior exam, consistent with the previous assumption of pseudoprogression given improved uptake. However, new metabolically active skeletal lesions were again seen. Interestingly, this time, again new lesions were seen mainly immediately adjacent to pre-existing lesions. We are not sure of the significance of the distribution of the new lesions. While findings could be reflective of progression, the overall pattern of changes in the osseous lesions is suggestive of possible ongoing pseudoprogression with continued infiltration of neoplastic lesions with activated immune cells. However, the patient was already four months from completion of sipuleucel-T treatment. Pseudoprogression with the possibility of the continued appearance of new lesions was described with F-18 FDG PET/CT for patients who continued to receive immunotherapy, which usually were checkpoint inhibitors that have a completely different mechanism of action. They also activate the immune system, but treatment response is usually seen as changes in measurable disease while on treatment. The sipuleucel-T treatment effect is observed much later and does not affect measurable disease but rather results in improved overall survival.

Given clinical deterioration and the presence of new lesions in imaging, the overall assessment was "true progression." The patient was started on chemotherapy.

The phenomenon of pseudoprogression related to the use of immunotherapy was extensively described in the literature for treatments with checkpoint inhibitors (for example, PD-1 targeting immunotherapy with pembrolizumab) [9]. Similarly to checkpoint inhibitors, sipuleucel-T is considered an active immunotherapy agent through its mechanism of activating a patient's immune system against a specific antigen. In essence, this represents a cancer vaccine by specifically targeting select antigens on the tumor cells. Checkpoint inhibitors are usually administered over long periods of time, months, and even years, allowing for continued immune response and multiple events of pseudoprogression over the entire time of treatment. Immune cells continue to attack occult lesions resulting in the initial appearance of new lesions or apparent enlargement of existing lesions with a subsequent robust favorable response. Sipuleucel-T is administered as a course of three injections two weeks apart. The measurable effects of this treatment were shown to be delayed. The phenomenon of pseudoprogression described for checkpoint inhibitors could theoretically be seen with sipuleucel-T as well, which is what we believed our case demonstrated. However, how long after the treatment could we suspect immune cell infiltration, and when will the increase in the size of the lesion start representing tumor growth and describing progression, requiring treatment adjustment? This will require further studies.

Patients must be identified who might benefit from continuing treatment beyond pseudoprogression on immunotherapy, and they must be distinguished from those who should have treatment changed due to true progression. However, there is another unique pattern of response related to immunotherapy, hyperprogression (HP), which is diagnosed based on evidence of accelerated disease progression related to immunotherapy and warrants immediate cessation of this particular immunotherapy [10]. Usually, a tumor burden increase of 1.5-folds or greater is suspicious for HP. For checkpoint inhibitors, the patient's performance status was very helpful in distinguishing between pseudoprogression, progression, or HP. Patients experiencing pseudoprogression usually have stable or improved performance status. With true progression and HP, patients tend to clinically deteriorate. Further studies are needed to understand why some patients rapidly progress despite treatment and how to identify true progression. The patient presented in this case felt well over the first several months after the immunotherapy, which enforced our assumption of the phenomenon of pseudoprogression rather than true progression. However, when clinical deterioration started about four months after immunotherapy, chemotherapy was started.

The Response Evaluation Criteria in Solid Tumors (RECIST 1.1), which is based on computed tomography (CT) and magnetic resonance imaging (MRI), has previously been validated and established for accurate radiological assessment. In 2009, it was first modified for immune-related Response Criteria (irRC) by using the WHO criteria in order to accurately gauge response to immunotherapy in clinical trials. Bi-dimensional irRC was later adapted to the uni-dimensional irRECIST (immune-related RECIST) criteria, allowing new tumor lesions to be considered [11]. Many variations of irRC and irRECIST have been proposed in the past decade, leading to inconsistency between studies. In attempts to establish standardized criteria, the official RECIST Working Group published the new iRECIST guideline in 2017 (Table 1). The most important change from RECIST 1.1 is that an additional follow-up is needed to confirm an ‘unconfirmed’ tumor progression after the initial increase in size [11].

**Table 1:** Definition of progressive disease in iRECIST vs RECIST 1.1 RECIST - Response Evaluation Criteria in Solid Tumors

	RECIST 1.1	iRECIST
New lesion	Represents progressive disease	No progression until confirmed
		Not added to tumor burden
Progressive disease (PD)	Increase 20% of the sum of long diameters compared to nadir (min 5mm)	Unconfirmed progressive disease (iUPD) if the patient’s performance status remains stable
	Or progression of non-target lesions	Confirmation of progression with imaging in 4-8 weeks after iUPD is diagnosed
	Or new lesion	
Confirmed PD	Confirmation is not required	Further increased size of the target or non-target lesions
		Increase in the sum of new target lesions >5 mm
		Progression of new non-target lesions
		Appearance of another new lesion

Prostate-specific antigen (PSA) is often used by clinicians in screening for prostate cancer. There are many options for imaging modalities to correlate with rising PSA levels. These include computed tomography (CT), magnetic resonance imaging (MRI), bone scan, and positron emission tomography (PET). However, many lack the necessary sensitivity to detect early disease progression, requiring high levels of PSA to be present. MRI of the pelvis and CT abdomen pelvis with contrast are unable to identify small metastatic lymph nodes. Bone scans have high sensitivity for osteoblastic metastases but both low sensitivity and specificity for osteolytic lesions, and cannot evaluate for tumor proliferation in the prostate, prostatectomy bed, extraprostatic extension, lymph nodes involvement, and presence of distant metastasis [12]. Given these limitations, PET imaging with novel radiotracers is offering a more promising solution. For example, 18F-fluciclovine (Axumin), which uses a leucine amino acid analog, has been shown to have high diagnostic accuracy in the detection of recurrent prostate cancer. The performance of fluciclovine PET is not dependent on a high PSA level. It can detect even small prostate cancer lesions at rather low PSA levels based on the demonstration of the presence of tumor cellular proliferation, with many studies demonstrating that it is able to detect lesions that other imaging modalities miss [12]. Moreover, the information from a fluciclovine PET could be used to define the field that will be surgically removed or treated with radiation therapy. This makes fluciclovine PET valuable for treatment planning, sometimes resulting in treatment modification such as widening the radiation field, identifying distant metastasis, or avoiding local radiation altogether [12].

Fluciclovine still has its caveats, showing non-specific uptake in infection, inflammation, benign prostatic hyperplasia, and some other malignancies, but it is better at characterizing lymph nodal and distant metastasis of prostate cancer, making it useful for early detection of recurrent prostate cancer. Immunotherapy causes infiltration of a tumor with immune cells which could result in increased accumulation of fluciclovine in the activated immune cells on PET. Patterns of uptake in inflammatory and infectious processes similar to those seen with F-18 fluorodeoxyglucose (FDG) were described, although evidence is still anecdotal. It is possible that in some cases, infiltration with immune cells could potentially manifest as increased intensity of F-18 fluciclovine uptake in previously already active lesions or development of new abnormal activity in previously occult lesions due to inflammatory reaction, similar to what is seen in imaging with FDG PET? Extensive literature exists describing increased FDG accumulation in areas of inflammation due to increased glucose utilization by immune cells. Our patient demonstrated a visually similar pattern of metabolic pseudoprogression with increased utilization of amino acids as demonstrated on fluciclovine PET. Further research is needed as to the utilization of amino acids by activated immune cells.

There is ongoing research on the identification of other potential imaging markers in metastatic prostate cancer. Prostate-specific membrane antigen (PSMA) is one such target that has shown promising results when compared to traditional imaging modalities. PSMA is overexpressed on the surface of prostate cancer cells. PSMA expression has been shown to have a positive correlation with more aggressive disease [13]. A 2016 study demonstrated evidence that PSMA-based imaging was able to detect suspected sites of prostate cancer that were not visualized or were unable to be differentiated on traditional CT imaging [14]. PSMA-targeting PET radiotracers were recently approved by FDA for use in the evaluation of patients with prostate cancer. Given the mechanism of binding of PSMA imaging compounds to a specific antigen on prostate cancer cells, immunotherapy-related pseudoprogression may have a different appearance on PSMA PET. An increase in the size of the lesions as a manifestation of pseudoprogression will probably still be seen on the CT portion of the PET-CT scan, but what metabolic changes we will see is still unknown. More research is needed in this direction.

It is important that radiologists and oncologists are aware of potential pseudoprogression and its appearance on a fluciclovine PET scan, as well as other different metabolic imaging modalities, and not mistake pseudoprogression for the true progression of the disease. It’s possible that sipuleucel-T therapy can cause the phenomenon of pseudoprogression on imaging given its mechanism of action, as demonstrated in our case, but further investigation of its effects is necessary.

## Conclusions

Metabolic imaging with PET-CT will continue to be a foundational tool when assessing for response to cancer treatment. As immunotherapy continues to become more widely utilized in treatment regimens, it is important for radiologists to understand phenomena such as pseudoprogression, and to recognize them in different metabolic imaging studies to prevent a false reading as disease progression, which can deleteriously lead to the discontinuation of an effective treatment. This case also reinforces the absolute necessity for ordering providers to include clinical notes, including current treatment regimens, for use by the diagnostic radiologist to provide crucial context to the patient presentation.
